# Prevention of exertional lower body musculoskeletal injury in tactical populations: protocol for a systematic review and planned meta-analysis of prospective studies from 1955 to 2018

**DOI:** 10.1186/s13643-018-0730-9

**Published:** 2018-05-05

**Authors:** Shawn D. Flanagan, Aaron M. Sinnott, Kellen T. Krajewski, Caleb D. Johnson, Shawn R. Eagle, Alice D. LaGoy, Meaghan E. Beckner, Anne Z. Beethe, Rose Turner, Mita Lovalekar, Courtenay Dunn-Lewis, Chris Connaboy, Bradley C. Nindl

**Affiliations:** 1 Neuromuscular Research Laboratory and Warrior Human Performance Research Center, Department of Sports Medicine and Nutrition, School of Health and Rehabilitation Sciences, University of Pittsburgh, Pittsburgh, PA USA; 2 Health Sciences Library System, University of Pittsburgh, Pittsburgh, PA USA; 3 Department of Health and Physical Activity, School of Education, University of Pittsburgh, Pittsburgh, PA USA

**Keywords:** Biomechanics, Epidemiology, Exercise, Prevention, Knee, Overuse, Performance, Rehabilitation, Risk factors, Training

## Abstract

**Background:**

Exertional lower body musculoskeletal injuries (ELBI) cost billions of dollars and compromise the readiness and job performance of military service and public safety workers (i.e., tactical populations). The prevalence and burden of such injuries underscores the importance of prevention efforts during activities necessary to sustain core occupational competencies. Attempts to synthesize prevention techniques specific to tactical populations have provided limited insight on the comparative efficacy of interventions that do not modify physical training practices. There is also a need to assess the influence of sex, exposure, injury classification scheme, and study design. Thus, the primary purpose of the systematic review and planned meta-analysis detailed in this protocol is to evaluate the comparative efficacy of ELBI prevention strategies in tactical populations.

**Methods:**

A systematic search strategy will be implemented in MEDLINE, EMBASE, Cochrane, and CINAHL. A multi-tiered process will be used to capture randomized controlled trials and prospective cohort studies that directly assess the prevention of ELBI in tactical population(s). Extracted data will be used to compare prevention strategies and assess the influence of heterogeneity related to occupation, sex, exposure, injury characteristics, and study quality. In addition, individual risk of bias, meta-bias, and the quality of the body of evidence will be rigorously tested.

**Discussion:**

This systematic review and planned meta-analysis will comprehensively evaluate ELBI mitigation strategies in tactical populations, elucidate factors that influence responses to treatment, and assess the overall quality of the body of research. Results of this work will guide the prioritization of ELBI prevention strategies and direct future research efforts, with direct relevance to tactical, health and rehabilitation science, and human performance optimization stakeholders.

**Systematic review registration:**

The systematic review protocol was registered with the International Prospective Register of Systematic Reviews (PROSPERO) on 3 Jan 2018 (registration number CRD42018081799).

**Electronic supplementary material:**

The online version of this article (10.1186/s13643-018-0730-9) contains supplementary material, which is available to authorized users.

## Background

The prevention of musculoskeletal injury (MSI) is central to efforts to improve the readiness, performance, and long-term health of tactical populations (i.e., military, police, firefighters, and emergency medical services (EMS)) [1–4]. In the US military, MSI is responsible for nearly 75% of limited duty cases and costs billions of dollars annually [5–9]. Physical exertion associated with training, occupational tasks, and recreation is the primary cause of acute hospitalization [10, 11], and 40% of such injuries occur in the lower body. The incidence of ELBI is similarly pronounced in police, firefighters, and EMS [12–16]. The burden of ELBI in tactical populations underscores the importance of injury prevention.

Reviews on ELBI prevention typically delimit injury assessment to a specific anatomic structure (e.g., groin, knee, ankle) [17–24] or classification (e.g., stress fractures, osteoarthritis, strains, sprains) [17, 20, 25–29]. The systemic effects of most interventions (e.g., additional or modified physical activity, educational programs, footwear modification) and integrative nature of structures within the lower kinetic chain justify a more generalized analysis of interventions and ELBI outcomes [30]. Additionally, a collective approach to the comparison of ELBI prevention strategies may improve translation to relevant stakeholders.

Despite growing evidence to support prevention strategies specific to tactical populations [8, 9, 31–38], current practices largely reflect information obtained from high school and college athletes [18, 19, 21, 23, 24, 39–43]. Most reviews emphasize subpopulations such as American football [27, 28, 41], soccer [19, 40, 44], or basketball [18, 20, 23, 24]. While relevant, the demands placed on tactical populations differ profoundly from athletics in terms of occupational tasks, exposure patterns, and underlying behavioral and environmental factors. In contrast to tactical populations for example, athletic physical exertion is generally defined, planned, and conducted in relatively well-controlled environments with predictable recovery periods.

A recent systematic review of military ELBI prevention identified six overarching strategies: (1) conditioning, (2) footwear modifications, (3) bracing, (4) physical activity volume, (5) physical fitness, and (6) leadership/supervision/awareness [30]. The majority of studies (26/43*)* sought to improve physical conditioning, but sex-specific responses were evident [30]. Less than half (19/44) of the interventions were specific to the military, and none examined public safety workers. A more comprehensive search strategy would likely improve the breadth of literature capture, especially in the case of novel or unusual interventions and less frequently studied tactical populations [45]. Furthermore, the stratification of aggregated data would improve analytic power and allow for normalized comparisons based on sex, exposure level, and injury classification [46].

In this systematic review and planned meta-analysis, we will identify and evaluate the collective prospective evidence on ELBI prevention in to tactical populations. The comparative efficacy of prevention strategies will be examined for ELBI overall, with additional sub-analyses of participant-, exposure-, and injury-specific responses determined by the extant literature base. The meta-analysis will use internationally recognized standards to assess individual and overall risk of bias, in addition to the quality of the body of evidence. Aggregate- and group-level data will be used to quantify the influence of study, participant, and injury characteristics. The findings of this review are intended to establish efficacy and support the prioritization of ELBI prevention strategies.

### Objectives

The primary objective of this systematic review and planned meta-analysis is to evaluate interventions and prospective observations for the prevention of ELBI in tactical populations. The proposed systematic review will address the following topics:

1. Comparative efficacy of intervention strategies to reduce ELBI in tactical populations.
2. Moderating influence of participant, study, and injury characteristics

## Methods

### Eligibility criteria

Studies will be selected based on the following criteria for design, participants, interventions, comparators, outcomes, and other study traits.

#### Study designs

To evaluate the comparative preventive efficacy of interventions for ELBI, controlled trials and prospective observational studies will be included. Acceptable study designs include parallel (between-group), cross-over (within-group), cluster, factorial trials, and non-randomized trials or prospective observational studies with direct between-group comparisons. Single-arm studies without direct comparisons will be excluded (e.g., benchmarking, simulated comparisons). Retrospective studies, cross-sectional studies, case reports and series, ideas, opinions, editorials, animal research, and in vitro research will be excluded.

#### Participants

Studies on adults aged 18 years or older will be included. Participants must be active military service members or public safety workers (including police, firefighters, and EMS). International military members that meet the national age requirement will be included. Studies of the target populations after retirement will be excluded.

#### Interventions

Qualifying studies will have the primary aim of preventing or mitigating ELBI in non-injured tactical population(s). Interventions targeting overall MSI risk will be included when lower body injury outcomes are presented or made available upon request. Non-specific behavioral, educational, or policy interventions will be included. Examples include new conditioning practices; modification or elimination of detrimental behaviors; education programs; policies that mandate, restrict, or otherwise influence occupational tasks; and external devices that prevent ELBI by providing protection or information.

Terminology to define injury is provided by the International Classification of Diseases (ICD-9-M) [47, 48] and National Institute for Occupational Safety and Health (NIOSH) [1]. Qualifying injuries will include fractures, derangements, dislocations, sprains, strains, and other injuries or disorders of the muscles, nerves, tendons, joints, or cartilage of the lower limbs. Such injuries must be caused, precipitated, or exacerbated by sudden or prolonged exertion involving movement repetition, force, vibration, or adverse biomechanics. Injuries resulting from falls, motor vehicle accidents, violence, and war will be excluded.

#### Comparators

Controls will include participants who receive placebo or “standard of care” treatment. The anticipated standard of care includes normal physical training activities without significant modification. Several potential comparators are relevant to the prevention of ELBI. Prominent examples are listed below but others will be considered on a case-by-case basis depending on the literature base.

1. Modification or elimination of practices that cause injury versus maintenance
2. High dose of intervention versus lower dose(s) of intervention
3. Interventions that influence conduct or access to activities associated with injury
4. Non-specific or multi-faceted behavioral, educational, or policy interventions versus no intervention or similar intervention(s) at a reduced dose

#### Outcomes

Outcomes and their definitions will be collected as reported in individual studies. Binary injury incidence data will be extracted to represent ELBI outcomes. Anticipated outcome measures include incidence, odds ratios, risk ratios, hazard ratios, and likelihood ratios. Outcomes may be derived from self-report, medical records, behavioral performance, medical imaging, physiological measurements, and biological specimens. Studies that do not report injury incidence will be excluded. Outcome measurements specific to ELBI will be extracted. Composite and upper body MSI will not be included, and studies that do not report or provide lower body-specific data will be excluded. If ELBI is subdivided by structure, tissue, category, or other factors, all levels of data will be extracted and pooled depending on injury classification.

#### Timing

Studies with a minimum surveillance period of 3 months will be prioritized. Due to many studies being conducted with recruits during basic training, to account for branches with shorter training periods [49, 50], studies with surveillance durations of at least 2 months will be considered if otherwise acceptable and of sufficient quality as determined by the review team.

#### Setting

Study setting is unrestricted.

#### Language

Included articles will be reported in English. When applicable, studies involving predominantly non-English speaking populations will be included.

#### Other review eligibility criteria

Study inclusion will not be restricted by geographic region. Inclusion will be limited to original research articles published in peer-reviewed journals. Abstracts, unpublished data, commentaries, letters, and conference proceedings will be screened. Data from 1955 to 2018 will be evaluated for systematic variation of effect estimates associated with publication age and normalized or sub-grouped as necessary.

### Information sources

MEDLINE, EMBASE, Cochrane, and CINAHL electronic databases will be searched. We will also search the reference lists of included publications, relevant reviews, and gray literature. Article listings in the top five journals of included studies will be searched from 2018 to 2003. The final reference list will be circulated among the review team and external experts identified by the team. The search strategy will meet IOM Standards for Systematic Reviews [51] and guidelines set forth in the Cochrane Handbook for Systematic Reviews of Interventions.

### Search strategy

The search strategy will use medical subject headings (MeSH) and text words that identify the target populations, injuries and structures, exposures and practices, and analysis features relevant to injury prediction. Searches will be limited to original human research articles published in peer-reviewed journals in English. Searches will not be limited by publication year or study design. The MEDLINE search strategy will be developed by a health sciences Librarian with expertise in systematic review searches (RT) in collaboration with the project team and reviewed by an additional Librarian (see Additional file 1 for search strategy). The MEDLINE search strategy will be adapted for use with EMBASE, Cochrane, and CINAHL databases. The electronic database search will be updated and re-run before the final analysis to capture new studies and confirm retrieval of a high proportion of eligible studies. PROSPERO will be searched for ongoing and completed reviews at the beginning and end of the review process. Recently completed systematic reviews will be searched for new articles.

### Study records

#### Data management

Citation abstracts and full-text articles captured during the literature search will be uploaded into Distiller Systematic Review (DSR) software for screening. Before DSR upload, duplicate search results will be removed using Endnote reference management software. When multiple reports on a study are identified, the study team will assess the consistency of reported study design, sample size, outcome(s), and statistical test(s). All relevant original information will be extracted and collated. In the event of multiple versions, the initial version will be retained. The inclusion and exclusion criteria will be developed into a series of forms for article screening (see Additional file 2 for screening forms). The forms will be tested and refined before formal screening. New members of the team will be trained on the content area and DSR software before participation.

#### Selection process

Titles of studies retrieved from the search will be independently screened by two reviewers for relevance to the prediction of ELBI. Reviewers will be blind to study author(s), journal title, and institution. For level 2 screening, abstracts will be reviewed for requisite study outcomes. Level 3 screening will confirm the eligibility of the participant population. Following the completion of level three screening, a quality control check will be conducted on all relevant articles by senior review team members (MB, KK, AL, AS) before the commencement of full-text screening. After the quality control check, if the inclusion criteria are met or inconclusive, the full text will be obtained for level four screening. Any article marked for removal will require agreement from three independent senior review members. When questions about study eligibility persist, study authors will be contacted for additional information. If the study author does not respond to contact for additional information or the response is deemed inadequate, the study will be screened and reason(s) noted. Disagreements about inclusion will be resolved through discussion or final review by a third author. Reason(s) for exclusion will be recorded and reported. Inter-rater agreement will be analyzed after the completion of three selection process procedures: (1) initial assessment test, (2) refinement, and (3) formal screening process.

#### Data collection process

Data will be extracted in duplicate by two independent reviewers using standardized forms in DSR. To improve consistency, data extraction forms will be pre-piloted in conjunction with the selection forms (see Additional file 3 for data extraction forms). Disagreements will be resolved by a third reviewer (SF, CC, CD, or ML). When data from primary studies are not presented in a usable format, the study authors will be contacted and asked to provide the missing data. We will attempt to contact primary authors up to three times by email or phone. The original study authors will also be contacted to confirm the accuracy of the final extracted data and resolve remaining uncertainties. In the event of uncertainty or missing data failure to respond, comply, or follow-up will result in study exclusion. When multiple reports of a single study are identified non-duplicated information will be collated. Otherwise, data from the initial report will be included with the exclusion of duplicates noted.

### Data items

Extracted data will include study characteristics (study setting and design, sample size, duration of follow-up, type of control, methods, intervention type, measurement techniques, time points, indicators of acceptability, side effects), participant and exposure characteristics (occupation, duration of participation, demographic, anthropometric, biological, workload, history, fitness), injury characteristics (injury definition, reporting method, location, and subtype,), author interpretation(s) of findings, suggested mechanisms of action, potential confounds, information for the assessment of risk of bias, and information consistent with the Template for Intervention Description and Replication (TIDieR) [52].

Outcomes specific to anatomic locations (e.g., knee), structures (e.g., anterior cruciate ligament), and disorders (e.g., sprain) will be collected for further analysis or aggregation. In the health and rehabilitation science literature, MSI is often classified as non-traumatic (overuse) or traumatic (acute). If descriptions are consistent across studies, injuries will be sub-typed accordingly. For cross-over trials, data from the first baseline period will be extracted. Measures of central tendency and variability will be extracted from figures when necessary using plot digitization software. If effect sizes cannot be calculated, study authors will be contacted for additional information.

### Outcomes and prioritization

#### Primary outcome

The primary outcome is ELBI incidence. Risk ratios will be calculated from the total number of participants at each measurement time point.

#### Secondary outcomes

1. Limited duty days prevented by the intervention
2. Cost reduction
3. Measures of acceptability, efficacy, and tolerability
  - Attrition for any reason: a measure of overall treatment acceptability
  - Attrition due to inefficacy of treatment: a global measure of efficacy
  - Attrition due to adverse events: a global measure of tolerability
4. Side effects
  - Number of participants who experienced one or more side effect

### Risk of bias

Two blinded reviewers will independently assess risk of bias for each included study. Disagreement between the reviewers will be resolved after discussion or final decision by a third reviewer (SF, CC, CD, or ML). To assess the risk of study bias and overall study quality, the following factors will be considered: sample definition and representation, adequate randomization, adequate allocation concealment, blinding, completeness of outcome data, and selective outcome reporting. For each domain, descriptions provided in the study will be collected as reported and scored using the risk of bias (RoB 2.0) for randomized controlled trials and the Risk of Bias In Non-randomized Studies - of Interventions (RoBINS-I) for prospective cohort studies [53]. When insufficient information is provided, the original study author(s) will be contacted for clarification. The risk of bias will be summarized graphically across studies. The synthesis will include a subgroup analysis to examine the influence of study quality on the overall findings of the review. Low-quality studies may be excluded from the final analysis.

### Data synthesis

Under the assumption of sufficient homogeneity, quantitative analysis of aggregate participant data will be conducted. The planned analytical approach will include a narrative synthesis of the findings from the included studies, structured around prevention interventions and participant, exposure, and study characteristics. If a study incorporates more than two *relevant* treatment groups, information from each group will be summarized with further analysis of combined data as appropriate (e.g., two levels of a treatment class combined for comparison against another treatment class). Original study authors will be contacted when there are missing data. Binary ELBI outcomes will be expressed as estimates of relative risk using risk ratios (lnRR) with 95% confidence intervals (CI).

The results of included studies will be pooled and analyzed using stratified random effects meta-analysis (RevMAN v5.3, The Cochrane Collaboration) [54, 55]. Subgroup and meta-regression (Stata, StataCorp) analyses will be used to determine whether treatment estimates are influenced by potential effect modifiers such as study characteristics and participant characteristics (see “Subgroup Analysis” section below). Statistical heterogeneity will be determined with chi-square (*χ*^2^) and *I*^2^ (0–100%) statistics, with *χ*^2^ *p* > 0.10 and *I*^2^ > 50% indicative of substantial heterogeneity [56]. Between-study variance (*τ*^2^) will be assessed in relation to effect size estimates. In the event of substantial heterogeneity [57], sensitivity analysis will be conducted as indicated by subgroup comparisons, RoB 2.0, and ROBINS-I checklists.

The presentation of results will be ordered by objective. Important comparisons and subgroup analyses will be presented based on the availability of data. Results will be presented in narrative text, tables, and figures. Depending on the results of sensitivity and subgroup analyses, studies with a high risk of bias may be excluded from the final meta-analysis.

#### Subgroup analysis

If the necessary data are available, subgroup analyses will be used to investigate sources of between-study heterogeneity and the robustness of the meta-analysis. Comparisons will be considered on a case-by-case basis depending on the literature base with the maximal feasible amount of data extracted for the following factors:

1. Participant characteristics (e.g., sex, age, occupation)
2. Injury location, structure, class (e.g., knee, patellar tendon, tendinopathy)
3. Injury subtype (acute vs. non-traumatic)
4. Intervention type (e.g., educational program vs. targeted training)
5. Surveillance period (e.g., 3 months vs. 1 year)
6. Study-specific factors
  - Study design (e.g., randomized vs. non-randomized)
  - Sample size
  - Overall study quality
  - Risk of bias (e.g., all studies versus low bias studies only)
  - Publication age

### Meta-bias

The possibility of publication and outcome reporting bias will be explored with funnel plots and the RoB 2.0 and ROBINS-I checklists. Small sample bias will be assessed by comparing fixed effects estimates with random effects models. Furthermore, continuous and dichotomous risk factors will be compared in RevMan and presented as interpretable rates (e.g., odds/risk/hazard ratio). Results may be used to re-weigh studies or for subgroup analysis. The analysis and reporting techniques used in this review will comply with guidelines provided by the Measurement Tool to Assess Systematic Reviews (AMSTAR-2) [58]. For information on compliance with PRIMSA-P review guidelines, see Additional file 4.

### Confidence in cumulative estimate

The overall quality of the body of evidence will be determined with the Grading of Recommendations Assessment, Development, and Evaluation (GRADE) approach [59]. Studies excluded from the meta-analysis will be excluded from the GRADE assessment. GRADE results will be used to inform conclusions on the overall strength of predictors of ELBI in military and public safety populations.

## Discussion

Exertional lower body musculoskeletal injuries negatively impact the health, performance, and readiness of military and public safety populations. There is a need to quantitatively synthesize and evaluate the comparative efficacy of strategies to prevent such injuries. In addition to a comprehensive search strategy, this systematic review and planned meta-analysis adds to the extant literature base in its use of rigorous multi-level screening processes and quantitative consideration of study bias, age, and quality. Prevention strategies with higher overall efficacy will be identified, with further analysis to examine the influence of participant-, injury-, and study-specific characteristics. The results of this review will provide a robust and comprehensive resource to guide the prioritization of ELBI prevention strategies and direct future research efforts.

## Additional files

Additional file 1: MEDLINE search strategy—terms and connectors for literature search. (PDF 25 kb)

Additional file 2: Screening form—questions and notes for each level of screening. (PDF 136 kb)

Additional file 3: Data extraction form—topics for data extraction, synthesis, and quality assessment. (PDF 113 kb)

Additional file 4: PRIMSA-P form—populated PRISMA-P checklist systematic review protocol. (PDF 290 kb)

## Abbreviations

AMSTAR-2: Measurement Tool to Assess Systematic Reviews
CI: Confidence intervals
CINAHL: Cumulative Index to Nursing and Allied Health Literature
DSR: Distiller Systematic Review
ELBI: Exertional lower body injury
EMBASE: Excerpta Medica Database
EMS: Emergency medical service
GRADE: Grading of Recommendations Assessment, Development, and Evaluation
ICD: International Classification of Diseases
IOM: Institute of Medicine
MEDLINE: US National Library of Medicine bibliographic database
MeSH: Medical subject headings
MSI: Musculoskeletal injury
NIOSH: National Institute for Occupational Safety and Health
PRISMA: Preferred Reporting Items for Systematic Reviews and Meta-Analyses
PROSPERO: International prospective register of systematic reviews
RCT: Randomized controlled trial
RoB-2.0: Revised tool for risk of bias in randomized trials
ROBINS-I: Risk of Bias In Non-Randomized Studies - of Interventions
RR: Risk ratio
TIDieR: Template for Intervention Description and Replication
