# Supplementary material for: Prevention of exertional lower body musculoskeletal injury in tactical populations: protocol for a systematic review and planned meta-analysis of prospective studies from 1955 to 2018
Source: Syst Rev. 2018 May 5;7:73. doi: 10.1186/s13643-018-0730-9 (PMC5936627; doi:10.1186/s13643-018-0730-9)
Supplement: Supplementary file 1 — MEDLINE search strategy—terms and connectors for literature search. (PDF 25 kb) [file 13643_2018_730_MOESM1_ESM.pdf]

ADDITIONAL FILE 1: Initial MEDLINE Search Strategy (Rose Turner, 12869 results)

("Athletes"[Mesh] OR "Athletic Injuries"[MeSH] OR "Sports"[Mesh] OR athlet*[tiab] OR badminton[tiab] OR baseball*[tiab] OR basketball*[tiab] OR bicycl*[tiab] OR bike*[tiab] OR bowling[tiab] OR cardiorespiratory fitness[tiab] OR cutting movement*[tiab] OR cyclist*[tiab] OR dance*[tiab] OR football*[tiab] OR golf*[tiab] OR gymnast*[tiab] OR handball[tiab] OR hockey*[tiab] OR judo[tiab] OR jump landing*[tiab] OR martial art*[tiab] OR rugb*[tiab] OR runner*[tiab] OR skiing*[tiab] OR snowboard*[tiab] OR soccer*[tiab] OR sport*[tiab] OR swim*[tiab] OR tennis[tiab] OR volleyball*[tiab] OR weightlift*[tiab] OR athlet*[ot] OR badminton[ot] OR baseball*[ot] OR basketball*[ot] OR bicycl*[ot] OR bike*[ot] OR bowling[ot] OR cardiorespiratory fitness[ot] OR cutting movement*[ot] OR cyclist*[ot] OR dance*[ot] OR football*[ot] OR golf*[ot] OR gymnast*[ot] OR handball[ot] OR hockey*[ot] OR judo[ot] OR jump landing*[ot] OR martial art*[ot] OR rugb*[ot] OR runner*[ot] OR skiing*[ot] OR snowboard*[ot] OR soccer*[ot] OR sport*[ot] OR swim*[ot] OR tennis[ot] OR volleyball*[ot] OR weightlift*[ot] OR "Firefighters"[Mesh] OR "Military Medicine"[Mesh] OR "Military Personnel"[Mesh] OR "Police"[Mesh] OR "Veterans"[Mesh] OR air force personnel[tiab] OR armed forces personnel[tiab] OR army personnel[tiab] OR army recruit*[tiab] OR coast guard[tiab] OR fire and rescue personnel[tiab] OR fire fighter*[tiab] OR firefighter*[tiab] OR infantry[tiab] OR law enforcement officer*[tiab] OR marines[tiab] OR military[tiab] OR navy personnel[tiab] OR police[tiab] OR sailor*[tiab] OR service member*[tiab] OR service personnel[tiab] OR soldier*[tiab] OR submariner*[tiab] OR troop*[tiab] OR veteran*[tiab] OR air force personnel[ot] OR armed forces personnel[ot] OR army personnel[ot] OR army recruit*[ot] OR coast guard[ot] OR fire and rescue personnel[ot] OR fire fighter*[ot] OR firefighter*[ot] OR infantry[ot] OR law enforcement officers[ot] OR marines[ot] OR military[ot] OR navy personnel[ot] OR police[ot] OR sailor*[ot] OR service member*[ot] OR service personnel[ot] OR soldier*[ot] OR submariners[ot] OR troop*[ot] OR veteran*[ot])

AND

("Arthralgia"[MeSH] OR "Arthritis"[MeSH] OR "Bone Diseases"[MeSH:noexp] OR "Bone Resorption"[MeSH] OR "Bursitis"[MeSH] OR "Compartment Syndromes"[MeSH] OR "Foot Deformities"[MeSH:noexp] OR "Foot Deformities, Acquired"[Mesh] OR "Foot Diseases"[Mesh:noexp] OR "injuries" [Subheading] OR "Joint Diseases"[MeSH:noexp] OR "Muscle Cramp"[MeSH] OR "Musculoskeletal Diseases"[MeSH:noexp] OR "Musculoskeletal Pain"[MeSH] OR "Patellofemoral Pain Syndrome"[Mesh] OR "Rhabdomyolysis"[MeSH] OR "Wounds and Injuries"[Mesh] OR dislocation*[tiab] OR fracture*[tiab] OR injuries[tiab] OR injury[tiab] OR pain syndrome*[tiab] OR sprain*[tiab] OR strain[tiab] OR strains[tiab] OR stress syndrome*[tiab] OR dislocation*[ot] OR fracture*[ot] OR injuries[ot] OR injury[ot] OR sprain*[ot] OR strain[ot] OR strains[ot]) AND ("Achilles Tendon"[Mesh] OR "Anterior Cruciate Ligament"[Mesh] OR "Bones of Lower Extremity"[Mesh] OR "Collateral Ligaments"[Mesh:noexp] OR "Foot Joints"[Mesh] OR "Gracilis Muscle"[Mesh] OR "Hamstring Muscles"[Mesh] OR "Hip Joint"[Mesh] OR "Knee Joint"[Mesh] OR "Lateral Ligament, Ankle"[Mesh] OR "Ligaments, Articular"[MeSH] OR "Lower Extremity"[Mesh] OR "Medial Collateral Ligament, Knee"[Mesh] OR "Muscles"[MeSH:NoExp] OR “Musculoskeletal System”[MeSH:NoExp] OR "Patellar Ligament"[Mesh] OR "Physical Education and Training"[Mesh] OR “Physical Fitness”[MeSH] OR "Plantar Plate"[Mesh] OR "Posterior Cruciate Ligament"[Mesh] OR "Psoas Muscles"[Mesh] OR "Quadriceps Muscle"[Mesh] OR "Round Ligament of Femur"[Mesh] OR “Running”[MeSH] OR ankle*[tiab] OR anterior cruciate ligament[tiab] OR collateral ligaments[tiab] OR femoral neck[tiab] OR femur*[tiab] OR foot[tiab] OR gracilis muscle[tiab] OR hamstring muscles[tiab] OR hip[tiab] OR hips[tiab] OR iliotibial band syndrome[tiab] OR knee*[tiab] OR leg[tiab] OR lower extremit*[tiab] OR lower limb*[tiab] OR medial tibial[tiab] OR meniscal[tiab] OR metatarsal*[tiab] OR patellar[tiab] OR patellofemoral[tiab] OR physical fitness[tiab] OR plantar plate[tiab] OR posterior cruciate ligament[tiab] OR psoas muscles[tiab] OR quadriceps muscle[tiab] OR shin splint*[tiab] OR shoes[tiab] OR vastus lateralis[tiab] OR ankle*[ot] OR anterior cruciate ligament[ot] OR collateral ligaments[ot] OR femoral neck[ot] OR femur*[ot] OR foot[ot] OR gracilis muscle[ot] OR hamstring muscles[ot] OR hip[ot] OR hips[ot] OR iliotibial band syndrome[ot] OR knee*[ot] OR leg[ot] OR lower extremit*[ot] OR lower limb*[ot] OR medial tibial stress syndrome[ot] OR meniscal[ot] OR metatarsal*[ot] OR patellar[ot] OR patellofemoral[ot] OR physical fitness[ot] OR plantar plate[ot] OR posterior cruciate ligament[ot] OR psoas muscles[ot] OR quadriceps muscle[ot] OR shin splint*[ot] OR shoes[ot] OR vastus lateralis[ot])

AND

("Epidemiology"[Mesh] OR "epidemiology" [Subheading] OR "Incidence"[Mesh] OR "Odds Ratio"[Mesh] OR "prevention and control"[Subheading] OR "Risk"[Mesh] OR cross product ratio[tiab] OR cross-product ratio[tiab] OR cross-product ratios[tiab] OR epidemiology[tiab] OR incidence[tiab] OR incidences[tiab] OR logistic regression*[tiab] OR occurrence*[tiab] OR odds ratios[tiab] OR predict*[tiab] OR prevention[tiab] OR preventive measures[tiab] OR preventive therapy[tiab] OR prophylaxis[tiab] OR relative odds[tiab] OR risk*[tiab] OR epidemiology[ot] OR incidence[ot] OR incidences[ot] OR logistic regression*[ot] OR occurrence*[ot] OR odds ratios[ot] OR predict*[ot] OR prevention[ot] OR preventive measures[ot] OR preventive therapy[ot] OR prophylaxis[ot])
