# Supplementary material for: Prevention of exertional lower body musculoskeletal injury in tactical populations: protocol for a systematic review and planned meta-analysis of prospective studies from 1955 to 2018
Source: Syst Rev. 2018 May 5;7:73. doi: 10.1186/s13643-018-0730-9 (PMC5936627; doi:10.1186/s13643-018-0730-9)
Supplement: Supplementary file 2 — Screening form—questions and notes for each level of screening. (PDF 136 kb) [file 13643_2018_730_MOESM2_ESM.pdf]

ADDITIONAL FILE 2: Initial Screening Form Questions by Level

Level 1- Title Screen

1. Does article evaluate the prediction and/or prevention of lower body musculoskeletal injuries in military service members, public safety workers, and athletes?

   a. Yes (to next question)
   b. Unsure (to level 2 screen)
   c. No (exclude)

Level 2- Abstract Screen

1. Does the current study describe injury <u>occurrence</u> in the methods, results, or discussion?

"Injury occurrence may be described in terms of: counts, frequency, incidence, prevalence, relative risk, odds ratios, likelihood ratios, risk ratios"

   a. Yes (to next question)
   b. No (exclude)
   c. Unsure-requires full text to answer (to next question)

Regardless, of injury occurrence, should investigation be excluded from analysis?

Note exclusion rationale:

- Not relevant to lower extremity injury occurrence/prevention
- Exclusively rehabilitation study
- Animal study
- Cadaver study/model simulation
- Pain study
- Not randomized control trial, prospective cohort, or case- control study. Exclude if study is a systematic review, narrative, meta-analysis, case-study, retrospective, or descriptive study
- Elderly/ geriatric

Level 3- Abstract Screen

1. Are participants active military, public service, or competitive athletes?

   a. Active Military
   b. Public service (police, firefighters, EMT, paramedics, prison guards)
   c. Competitive athletes
   d. Unsure (need full text to answer)
   e. No. Study population not relevant (exclude)

Level 4- Full Text Screen

1. Is the investigation relevant to the study purpose and should it be included in the analysis?

   a. Yes, it is a relevant study and should be included.
   b. No, it is irrelevant (note exclusion rationale)
   c. Not relevant to lower extremity injury occurrence/prevention
   d. Exclusively rehabilitation study
   e. Animal Study
   f. Cadaver study/ model simulation
   g. Pain study
   h. Study is a systematic review, narrative, meta-analysis, retrospective, or descriptive study
   i. Elderly/geriatric
   j. Exclusively injuries resulting from falls, car accidents, explosions, and other scenarios unrelated to physical training

2. Is the current study original research and describe injury occurrence in the methods, results, or discussion?

    a. Yes, clearly described.
    b. Not clearly described. Need to contact author for more information
    c. No description, and does not indicate occurrence, (exclude)

3. Are participants’ active military, public service, or competitive athletes?

    a. Military or Public Safety Workers (police, fire, EMT, paramedic, prison guard)
    b. Competitive athletes.
    c. Not clearly described, need to contact author.
    d. No. study population not relevant (exclude)

4. What is the study design?

    a. Randomized controlled trial
    b. Quasi-randomized controlled trial
    c. Prospective comparative cohort study
    d. Case control or nested case control study (exclude)
    e. Other design (exclude)

5. Are participants 18 years of age or older?

    a. Yes (include)
    b. No, some participants are under and over 18 (exclude)
    c. No (exclude)

6. Did the study design include an observation length of 12 weeks or longer?

    a. Yes. Study was 12 weeks or longer (include)
    b. No, duration 8-11 weeks
    c. No Less than 8 weeks (exclude)
    d. Information not provided (include)
