# Supplementary material for: Prevention of exertional lower body musculoskeletal injury in tactical populations: protocol for a systematic review and planned meta-analysis of prospective studies from 1955 to 2018
Source: Syst Rev. 2018 May 5;7:73. doi: 10.1186/s13643-018-0730-9 (PMC5936627; doi:10.1186/s13643-018-0730-9)
Supplement: Supplementary file 3 — Data extraction form—topics for data extraction, synthesis, and quality assessment. (PDF 113 kb) [file 13643_2018_730_MOESM3_ESM.pdf]

ADDITIONAL FILE 3: Data Extraction Items

1. Study Characteristics

a. Setting
b. Study Design
c. Sample Size
d. Duration of follow-up/surveillance period
e. Type of control
f. Methods and intervention details
g. Measurement techniques
h. Measurement time points

2. Potential risk factors

a. Occupation
b. Duration of service/participation/deployments
c. Demographic, Anthropometric, and Biological
   i. Sex
   ii. Age
   iii. Race
   iv. Height
   v. Somatotype
   vi. Body composition (fat mass, lean mass, fat percentage, body mass index)
   vii. Bone composition (density, mass)
d. Workload
   i. Running mileage
   ii. Dosage and timing of activity exposure
   iii. Load carriage magnitude and frequency
   iv. Footwear
e. Personal History
   i. Physical activity
   ii. Previous injury
   iii. Smoking
   iv. Medication use
   v. Vitamin D physiology
   vi. Sleep quality/quantity
   vii. Mental/behavioral health
f. Physical Fitness
   i. Aerobic capacity
   ii. Anaerobic capacity

iii. Flexibility
iv. Maximal voluntary dynamic or isometric muscular strength
v. Maximal muscular power
vi. Movement Screen (e.g. FMS)
vii. Muscular Endurance

3. Outcomes
a. Injury characteristics
i. Definition
ii. Structure(s) or location(s)
iii. Disorder(s)
iv. Classification (e.g. overuse vs. acute)
b. Effect of intervention(s) on limited duty days
c. Effect of intervention(s) on healthcare utilization or employment costs
d. Indicators of acceptability, efficacy, and tolerability
i. Attrition for any reason (acceptability)
ii. Attrition due to inefficacy of treatment (efficacy)
iii. Attrition due to adverse events (tolerability)
e. Side effects
i. Number of participants who experienced one or more side effect

4. Primary study conclusions

5. Suggested mechanisms to explain relationship risk factors and injury

6. Potential confounds

7. Items to complete the following checklists:
a. Risk of Bias (ROB 2.0) for RCTs and Risk of Bias in Non-randomized Studies – of Interventions (ROBINS-I)
b. Grading of Recommendations Assessment, Development, and Evaluation (GRADE)
